# Neither Chia Flour nor Whey Protein Supplementation Further Improves Body Composition or Strength Gains after a Resistance Training Program in Young Subjects with a Habitual High Daily Protein Intake

**DOI:** 10.3390/nu15061365

**Published:** 2023-03-11

**Authors:** Hermann Zbinden-Foncea, Claudia Ramos-Navarro, Victoria Hevia-Larraín, Mauricio Castro-Sepulveda, Maria José Saúl, Cesar Kalazich, Louise Deldicque

**Affiliations:** 1 Exercise Physiology and Metabolism Laboratory, School of Kinesiology, Faculty of Medicine, Universidad Finis Terrae, Santiago 7500000, Chile; hzbinden@uft.cl (H.Z.-F.); cramosn@uft.edu (C.R.-N.); victoriahevia@gmail.com (V.H.-L.); mcastro@uft.cl (M.C.-S.); mjsaul@hotmail.com (M.J.S.); 2 Centro de Salud Deportiva, Clínica Santa María, Santiago 7571894, Chile; 3 Institute of Neuroscience, UCLouvain, 1348 Louvain-la-Neuve, Belgium; 4 Faculty of Health Science, Universidad Francisco de Vitoria, 28223 Madrid, Spain; 5 Clínica MEDS, Santiago 7571894, Chile; cesar.kalazich@gmail.com

**Keywords:** plant-based protein, resistance training, fat-free mass, omega-3

## Abstract

The aim of this study was to compare the potential additional effect of chia flour, whey protein, and a placebo juice to resistance training on fat-free mass (FFM) and strength gains in untrained young men. Eighteen healthy, untrained young men underwent an 8-week whole-body resistance training program, comprising three sessions per week. Subjects were randomized into three groups that after each training session consumed: (1) 30 g whey protein concentrate containing 23 g protein (WG), (2) 50 g chia flour containing 20 g protein (CG), or (3) a placebo not containing protein (PG). Strength tests (lower- and upper-limb one repetition maximum (1 RM) tests) and body composition analyses (dual-energy X-ray absorptiometry; DXA) were performed before (PRE) and after (POST) the intervention. Resistance training increased FFM and the 1 RM for each of the strength tests similarly in the three groups. FFM increased by 2.3% in WG (*p* = 0.04), by 3.6% in CG (*p* = 0.004), and by 3.0% in PG (*p* = 0.002)., and 1 RM increased in the different strength tests in the three groups (*p* < 0.05) with no difference between PG, CG, and WG. In conclusion, neither chia flour nor whey protein supplementation elicited an enhanced effect on FFM and strength gains after an 8-week resistance training program in healthy, untrained young men consuming a habitual high protein mixed diet (>1.2 g/kg/day).

## 1. Introduction

Muscle mass is essential for health and sport performance. Muscle mass is tightly regulated by muscle protein synthesis (MPS) and muscle protein breakdown. The main stimuli for MPS are amino acid availability and resistance training, which synergically affect MPS, contributing to muscle mass accrual over time [1,2,3]. The magnitude of the increase in MPS in protein-matched sources is generally greater in response to animal-derived proteins [4,5]. Plant-based proteins generally have a less favorable essential amino acid profile, especially for leucine, than animal-based proteins [6] and have a reduced digestibility [7], which affects amino acid delivery to the skeletal muscle [6,7,8].

Protein supplementation has repeatedly been demonstrated to enhance lean mass and strength gains after resistance training in healthy young subjects with an optimal daily protein intake around 1.6 g/kg/d [9]. Several studies investigated the potential for plant vs. animal protein sources to increase muscle mass after resistance training. Most studies used whey or milk protein as the animal protein source and soy protein as the plant protein source, but conflicting results were obtained [10,11]. An interesting plant-based protein source is chia seed. The consumption of chia has been increasing over the years due to its health-related nutritional components, including high essential fatty acids, dietary fibers, proteins, antioxidants, and other micronutrients [12,13]. Although, like other plant proteins, the amino acid profile of chia flour presents a lower leucine and essential amino acid (EAAs) content than whey protein, chia flour contains all EAAs and has a greater digestibility than chia seeds [12]. Moreover, chia has a high omega-3 polyunsaturated fatty acid content (*n*-3), which may improve muscle function and lead to a higher *n*-3 composition of muscle tissue phospholipids [14]. *n*-3 supplementation has been found to increase MPS and to support muscle mass accrual over time [15,16]. To date, no study has investigated the effect of chia flour supplementation as a plant protein source rich in *n*-3 on the chronic adaptive responses to resistance exercise.

Here, we hypothesized that chia flour supplementation would enhance the effects of a resistance training program on fat-free mass (FFM) and strength gains to the same extent as whey protein, due to its high protein and *n*-3 content, in untrained young men. Therefore, we aimed to compare the effects of combining an 8-week whole-body resistance training program with a placebo, chia flour, or whey protein supplementation on FFM and strength gains in untrained young men.

## 2. Materials and Methods

### 2.1. Participants 

Eighteen healthy untrained young males (age, 22.4 ± 3.1 y; body weight (BW), 66.6 ± 6.8 kg; height, 1.78 ± 0.04 m; body mass index (BMI), 22.3 ± 1.9 kg/m^2^) participated in the study. First, the suitability of the subjects was assessed by questionnaire. The inclusion criteria were males between 18 and 30 y, not suffering from any metabolic disease, BMI ≤ 25 kg/m^2^, and no previous resistance training experience. The exclusion criteria were regular exercise performed >3 times/week, use of dietary supplements, and any muscle injuries. The study was approved by the Ethics Committee from the Universidad Finis Terrae, and all subjects signed an informed consent form prior to actively participating in the study. The study was conducted according to the principles of the Declaration of Helsinki.

### 2.2. Experimental Design

This was a randomized, controlled, single-blinded study. Eighteen participants were randomized into three groups: a placebo group (PG; *n* = 6), a chia flour supplement group (CG; *n* = 6), and a whey protein supplement group (WG; *n* = 6). Subjects were randomized according to anthropometric characteristics (weight, height, and BMI). An independent researcher assigned the subjects to their respective groups using the sealed envelope system. The intervention consisted of 8 weeks of resistance training, performed three times a week, and the consumption of either a placebo, a whey protein supplement, or a chia flour supplement after each training session, in liquid form (see below). Participants were not informed about the content of the drink they received. Strength and body composition were assessed at baseline and upon completion of the 8-week training period. Similarly, energy intake and macronutrient distribution were assessed via a three-day recall food diary at baseline and at study completion. Subjects were instructed not to change their usual diet throughout the study and were monitored by a certified dietitian. 

### 2.3. One Repetition Maximum Protocol 

One-repetition maximum (1 RM) assessment was performed for the following exercises: chest press, shoulder press, pull down, seated row, horizontal leg press, inclined leg press, and leg extension. For each exercise, a warm-up of 8–10 repetitions at approximately 50% of the estimated load achieved in the first 1 RM attempt was performed. This warm-up was also used to familiarize the subjects with the testing equipment and lifting techniques the week before the testing day. The testing procedure was initiated 2 min after the warm-up. The participants selected the amount of weight to lift according to their previous effort and were instructed to use enough weight to perform a maximum of five repetitions (set at approximately 70% of their predicted 1 RM). After 1–2 min rest, participants were asked to perform 3 repetitions with a load set at approximately at 80% of their predicted 1 RM. After increasing the load and 1–2 min of rest, the participants were asked to lift the weight in one attempt. If further attempts were needed for weight adjustment, participants were given 3–5 min rest between each attempt [17]. The 1 RM was recorded as the final weight lifted for which the subject completed only one single maximal execution. 

### 2.4. Resistance Training

All exercise training sessions were performed at the Finis Terrae University gym (Santiago, Chile) and supervised by a physical therapist. All participants were instructed on correct weight lifting techniques to ensure correct execution before starting the whole-body resistance training program. Each training session started with a light 10 min warm-up on a static ergometer. The resistance training program consisted of a progressive protocol. At the beginning of each session, all training program exercises were performed with light resistance for 20 repetitions. Each workout included the same exercises as for the 1 RM tests (see above) and were repeated 3 times per week for a total of 8 weeks. Training intensity was set at 75% of 1 RM of each subject measured at baseline. Subjects performed 3 sets of 8–12 repetitions for each exercise, with 1–2 min rest. As soon as the subjects were able to exceed the number of repetitions in the 3 sets, the resistance was increased by 2 kg for each exercise. Thereby, a progressive load increase was achieved up to week 4. At week 4, a new 1 RM test was performed, and the load of each exercise was adjusted for the remaining 4 weeks of training. At the end of the whole-body resistance training program, all participants performed one last 1 RM test for each exercise to determine overall changes in strength.

### 2.5. Dietary Supplementation 

The chia flour, whey protein, or placebo drinks were consumed within 10 min after each training session. The nutritional composition of the drinks is detailed in Table 1. The chia flour drink was a type of smoothie composed of 50 g Chia Powder (XIA-125, Benexia^®^), vanilla, and artificial sweetener (Gourmet^®^) diluted in 600 mL of water. It contained a total of 20.1 g protein and 3 g *n*-3. The whey protein drink was a type of smoothie composed of 30 g whey protein powder (Syntrax^®^) diluted in 250 mL of water. It contained 23 g protein. The placebo drink was sugar-free orange juice (Daily^®^), diluted in 600 mL water. Compliance and tolerance of the drinks were assessed using a survey at the end of the study.

### 2.6. Tolerance and Acceptance Survey 

Subjects completed a survey at the end of the study (week 8) to assess tolerance of the supplements in terms of organoleptic characteristics (smell, taste, aspect) and the occurrence of any gastrointestinal symptoms (vomiting, diarrhea, stomachache, constipation). Furthermore, changes in appetite and satiety were assessed using a Likert scale as previously published [18]. 

### 2.7. Body Composition 

Body composition was determined at PRE and POST (2 days after the last training session in the morning) using whole body dual-energy X-ray absorptiometry (DXA, GE Healthcare, Madison, WI, USA). DXA scans were used to determine changes in body composition, BW, fat mass (FM), and FFM.

### 2.8. Food Intake Records and Dietary Analysis

At PRE and POST, participants completed a 3-day dietary record to estimate total habitual energy (kcal/day) intake, macronutrient intake, and fiber intake. Participants were instructed by a certified dietitian on procedures involving food records and were asked not to modify their diet throughout the whole study. The participants recorded their diet themselves for 3 days over 1 week (2 working days and 1 weekend day) to represent their usual food consumption. For dietary analysis, food diaries were logged into the Food Processor 7.6 (ESHA Nutrient Analysis software) [19]. 

### 2.9. Statistical Analysis

Sample size was estimated based on an effect size of 2.3, an alpha level of 0.05, and a power of 0.97 (G * Power 3.1, Germany), which indicated that 5 participants per group would suffice. Those values were based on Taylor et al. (2016), who tested the effects of whey protein supplementation and anaerobic and resistance training on trained female athletes [20]. Statistical analysis was performed using GraphPad prism v.6.0 (San Diego, CA, USA). The Kolmogorov–Smirnov test was used to check the normality distribution. Baseline characteristics and comparisons between groups were determined using a one-way ANOVA. Two-way repeated measured analysis of variance (ANOVA) was used to determine any group-by-time interaction in body composition, dietary intake, and strength assessments. All results are presented as means ± standard deviation (SD). Delta values (Δ) are shown as means and 95% confidence interval (Δ 95% CI). The alpha level of significance was set at *p* ≤ 0.05.

## 3. Results

### 3.1. Estimated Dietary Intake

Estimated dietary intake as obtained from a three-day dietary record is shown in Table 2. No group-by-time interaction effect was found in either energy or macronutrient ingestion before or after the study. Noteworthy in the context of the present study is the habitual daily protein intake of the participants. In PG, the daily protein intake was 1.3 ± 0.2 g/kg/d, in CG it was 1.5 ± 0.4 g/kg/d, and in WG it was 1.5 ± 0.3 g/kg/d, with no difference between the three groups.

### 3.2. Supplementation and Tolerance Survey 

According to the survey carried out at the end of the study, all participants consumed their respective supplements after each training session. Upon study completion, participants were asked to guess which supplement they consumed. No participant correctly guessed the supplement they had received. Adverse effects were reported by the CG and WG groups (Table 3). In CG, the most common was abdominal distension (67% of the participants), diarrhea (17%), and meteorism (17%). Furthermore, all participants in CG reported satiety for 2 hours after supplement ingestion. In WG, only one participant reported abdominal distention (17%) and four participants reported satiety post-ingestion (67%). No adverse effects were found in PG. 

### 3.3. Strength 

1 RM values for each exercise are shown in Table 4. No statistical differences were observed between groups at PRE. At POST, all groups improved their 1 RM for each muscle group tested (*p* < 0.05) compared to baseline. However, no group-by-time interaction effect was observed for any of the assessed exercises.

### 3.4. Body Composition 

No statistical differences were observed between groups at PRE for any body composition variable (Table 5). FFM increased in all three groups at POST as follows: by 2.3% in WG (*p* = 0.04), by 3.6% in CG (*p* = 0.004), and by 3.0% in PG (*p* = 0.002). No group-by-time interaction effect was detected. 

## 4. Discussion

The present study aimed to compare the potential additional effect of chia flour, whey protein, and a placebo juice to an 8-week whole-body resistance training program on FFM and strength gains in untrained young men. Similar increases in FFM and strength were observed following the intervention in all three groups. The present study emphasizes the following points related to resistance training and protein supplementation: (1) optimal protein intake, i.e., 1.3–1.4 g/kg/d, ensures chronic adaptive responses to resistance training; (2) addition of plant or animal protein to a high protein mixed diet does not result in additional muscle anabolism in untrained men; (3) chia flour as a source of protein and *n*-3 are insufficient to further improve muscle mass and function after resistance training in untrained young men with a habitual high protein intake.

Chia is a high-protein plant source with a high amino acid quality profile compared to other plant proteins and is easily digestible [12]. Despite many studies comparing the anabolic effects of plant vs. animal protein, chia has never been investigated so far. Most studies compared the anabolic effects of soy versus whey protein or milk using diverse resistance training protocols. In one of the first studies, a placebo, a 1.2 g/kg/day soy supplement, and a 1.2 g/kg/day whey supplement induced increases in FFM and strength after 6 weeks of resistance training, but the gains were higher in the two protein-supplemented groups than in the placebo group, with no difference between soy and whey protein [10]. In another study, FFM gains after 9 months of resistance training were higher when 21 g whey protein were provided compared to 20 g soy protein or a placebo [21]. Similarly, FFM gains after 12 weeks of resistance training were higher after milk ingestion than after a soy drink or a placebo after each training session, with both milk and the soy drink providing 17.5 g protein [11]. In response to an 8-week resistance training program, no additional effect of 48 g/day pea protein [22] or 48 g rice protein after each of the 3 training sessions per week was found on FFM and strength gains compared to whey protein [23]. However, no placebo group was included in the latter two studies, and the dietary protein intake was not controlled throughout the interventions. Collectively, those studies are ambiguous on the anabolic effects of a relatively small range of plant- vs. animal-based protein supplements.

Here, we did not find any additional effect of either chia or whey protein on FFM and strength gains after 8-week resistance training compared to a placebo. The lack of effect of our two protein supplements might be explained by sufficient EAAs and leucine provision for optimizing MPS after resistance exercise through the habitual high protein diet in the present study. Of note, the recruitment of the participants was not based on the protein content in their diet. The high protein content of 1.3–1.4 g/kg/d reflects the usual protein intake in young active but not specifically resistance-trained men in Chile. This high protein intake was found after the completion of the study when the dietary record was analyzed for each participant. This high daily protein intake could have limited and even masked the potential effects of chia and whey supplementation, a phenomenon described as the muscle full hypothesis [24]. Indeed, MPS responds in a dose-dependent but saturable fashion to the levels of circulating EAAs [24]. Here, it is possible that the habitual diet already provided a saturable amount of EAAs. This said, it was previously found that a soy and a whey supplement of 1.2 g/kg/day induced greater anabolic effects compared to a placebo after a 6-week resistance training in participants having a daily protein intake above 1.6 g/kg, excluding protein supplements [10]. Thus, it seems that the muscle full hypothesis is not always verified. 

In addition to being rich in protein, chia presents a high *n*-3 polyunsaturated fatty acid content that has been suggested to be an interesting nutritional strategy to promote muscle and strength gains [25]. *n*-3 enhances MPS rates by still unknown mechanisms that seem to depend on the presence of eicosapentaenoic acid (EPA) and docosahexaenoic acid (DHA) in myocyte membranes. This may improve amino acid uptake and thereby enhance MPS [16,26]. However, data in this regard are still conflicting [16,27,28]. In a chronic setting, supplementation of 4 g *n*-3/d from fish oil improved body composition after a 6-week intervention in young individuals, which was not combined to exercise training [29]. To the best of our knowledge, only one study has investigated the combined effect of a higher-protein diet with *n*-3 supplementation and resistance training in healthy untrained females [30]. The study suggested no benefit of *n*-3 supplementation on FFM gains. However, the trial consisted of a relatively short, i.e., 4-week, resistance training period. With chia being the richest source of plant-based *n*-3 [31] and a rich source of plant-based protein, we found it interesting to investigate whether chia flour could improve muscle anabolism and strength after a longer resistance training program of 12 weeks.

To the best of our knowledge, only one study has investigated the combined effect of a high-protein diet and *n*-3 after resistance training in healthy untrained subjects [30]. The study suggested no benefit of protein and *n*-3 supplementation on FFM gains. However, the trial consisted of a relatively short duration resistance training protocol of 4 weeks. As during the first weeks of resistance training edema might contribute to the gain in FFM, with a limited accrual of myofibrillar proteins [32], the study design prevented conclusions being drawn regarding the anabolic response to a higher-protein diet combined with *n*-3 after resistance training. In addition, there was no group supplemented with protein only, which made any conclusion on the additional effects of omega-3 difficult.

Here, this is the first study comparing the effects of chia flour as a plant-based supplement rich in protein and *n*-3 to a placebo and to whey protein in young men undergoing a whole-body resistance training program for 8 weeks. We did not observe differences in the anabolic effects between chia flour, whey protein, and placebo, suggesting that the resistance training per se was sufficient to induce an adequate anabolic response in subjects consuming a high protein intake of ~1.3–1.4 g/kg/d. Those results are consistent with other studies suggesting that the total daily protein intake determines muscle mass and strength gains rather than the protein source [33,34]. In addition, we dismiss the idea that chia might have an additive anabolic and functional advantage despite its high *n*-3 content, which has been associated with improved muscle function [14]. The dose of *n*-3 cannot explain the lack of additive effect of chia provided here, i.e., 3 g, as it was close to the 5 g used in the studies that demonstrated an increase in *n*-3 composition in muscle tissue [35]. What should be mentioned, though, is that *n*-3 in chia is found in the form of alpha-linolenic acid (ALA), which requires an endogenous conversion to both EPA and DHA to be metabolically active in humans. However, the biochemical pathways in humans that convert ALA to EPA and DHA are limited [36], possibly resulting in lower incorporation of *n*-3 from chia into muscle cells compared to *n*-3 from fish oil, which does not require this conversion. Further investigation should determine whether *n*-3 from plant sources is less efficient than that from animal sources on muscle anabolism and function.

Our study is a novel, well-controlled clinical trial and the first to investigate the anabolic effects of chia flour after a resistance training program in young untrained males. However, this study is not without limitations. First, we did not assess *n*-3 intake from dietary sources. Therefore, it is impossible to know the total daily amount of *n*-3 in the present study. This said, any external supplementation in the present study, amongst which was *n*-3, was part of the exclusion criteria. Second, our results are limited to body composition as we did not include other muscle size measurements, such as muscle or fiber cross-sectional area. Nonetheless, DXA scans are used as a reference technology with low precision error for assessing lean mass as a proxy of muscle mass [37]. Finally, we did not directly assess the physical activity level of the subjects. Inclusion in the study was based on the number of exercise sessions per week, which had to be less than three times per week. The conclusions of the present study may thus only be extended to that specific population.

## 5. Conclusions

Neither chia flour nor whey protein further improved FFM and strength gains induced by an 8-week resistance training program in healthy untrained young men with a habitual high protein mixed diet (>1.2 g/kg/day). Further investigations are warranted to test the efficiency of chia flour in populations with lower protein intake, such as vegetarians, vegans, and the elderly.

## Figures and Tables

**Table 1:** Nutritional information for the chia flour, whey protein and the placebo juice.

Nutritional Information	Chia Flour (50g)	Whey Protein (30g)	Placebo Juice
Energy (kcal)	128	120	0
Total carbohydrates (g)	18.2	3	0
Dietary fiber (g)	17.6	0	0
of which soluble (g)	4.05	0	0
of which insoluble (g)	13.2	0	0
Total sugar (g)	0.6	1	0
Total protein (g)	20.1	23	0
Leucine (g)	1.3	2	0
Total fat (g)	5.0	1.5	0
Saturated fat (g)	0.6	0.5	0
Monounsaturated fat (g)	0.4	1	0
Polyunsaturated fat (g)	4.0	0	0
of which linoleic acid (g)	0.97	0	0
of which alpha-linolenic acid (g)	3.0	0	0

**Table 2:** Dietary energy and macronutrient intake before (PRE) and after (POST) an eight-week resistance training program in the placebo (PG), chia flour (CG) and whey protein (WG) group.

		Energy Intake (kcal/day)	Protein Intake (g/day)	Protein Intake (g/kg/day)	Fat Intake (g/day)	Fat Intake (g/kg/day)	CHO Intake (g/day)	CHO Intake (g/kg/day)
PG	PRE	2193 ± 403	87 ± 8	1.3 ± 0.2	79 ± 16	1.2 + 0.3	283 ± 60	4.4 + 1.2
(*n* = 6)	POST	2136 ± 516	98 ± 14	1.5 ± 0.3	81 ± 19	1.210.3	249 ± 80	3 ± 1.4
	∆	−57 ± 223	12 ± 12	0.1 ± 0.2	2 ± 15	−3.1 ± 0.2	−34 ± 40	−0.6 ± 0.7
	∆ 95% CI	−290 − 177	−0.6 − 24	−0.1−0.4	−13 − 18	0.2 − 0.2	−76 − 7	−1.4 − 0.1
	*p*-value	0.95	0.6	0.66	0.96	0.99	0.96	0.41
CG	PRE	23591309	105 ± 34	1.5 ± 0.4	77 ± 26	1.1 ± 0.4	307 ± 36	4.6 ± 0.7
(*n* = 6)	POST	2048 ± 581	121 ± 52	1.8 ± 0.7	64 ± 15	0.9 ± 0.2	263 ± 67	3.9 ± 0.8
	∆	−311 ± 413	17 ± 31	0.2 ± 0.5	−13 ± 20	−0.2 ± 0.3	43 ± 98	−0.7 ± 1.4
	∆ 95% CI	−745 − 123	−16 − 49	−0.2 − 0.7	−34−8	−0.5 − 0.1	−145 − 60	−2.1 − 0.8
	*p*-value	0.15	0.3	0.30	0.13	0.17	0.13	0.33
WG	PRE	2088 ± 580	102 ± 28	1.5 ± 0.3	63 ± 22	0.9 ± 0.3	269 ± 91	4.0 ± 1.2
(*n* = 6)	POST	2173 ± 657	108 ± 39	1.6 ± 0.5	67 ± 19	1.0 ± 0.2	279 ± 99	4.2 ± 1.4
	∆	84 ± 419	5 ± 25	0.1 ± 0.3	3 ± 7	0.1 ± 0.1	9 ± 6	0.2 ± 0.9
	∆ 95% CI	−356 − 525	−21 − 33	−0.2 − 0.4	−4 − 12	−0.1 − 0.2	−59 − 79	−0.8 − 1.1
	*p*-value	0.92	0.92	0.93	0.90	0.90	0.90	0.97
Interaction *p*-value		0.2	0.74	0.72	0.12	0.18	0.41	0.31

Values are presented as mean ± standard deviation (SD), with all delta values (∆) and 95% confidence interval from delta values (∆ 95% Cl). Interaction *p*-value, 3 groups (PG, CG and WG) × 2 times (PRE and POST).

**Table 3:** Summary of adverse events and satiety in the placebo (PG), chia flour (CG), and whey protein (WG) groups.

Adverse Event	PG (*n* = 6)	CG (*n* = 6)	WG (*n* = 6)
Abdominal distension	0	4	1
Diarrhea	0	1	0
Meteorism	0	1	0
Satiety post ingestion	0	6	4

**Table 4:** Maximal strength measurements before (PRE) and after (POST) an 8-week resistance training program in the placebo (PG), chia flour (CG), and whey protein (WG) group.

Groups		Chest Press (kg)	Shoulder Press (kg)	Pull Down (kg)	Seated Row (kg)	Horizontal Leg Press (kg)	Leg Press (kg)	Leg Extension (kg)
PG (*n* = 6)	PRE	59 ± 13	43 ± 12	40 ± 9	51 ± 10	71 ± 15	165 ± 36	84 ± 12
	POST	75 ± 12	57 ± 12	49 ± 12	62 ± 14	113 ± 30	224 ± 33	100 ± 16
	Δ	15 ± 8	14 ± 7	8 ± 5	11 ± 6	42 ± 19	59 ± 30	16 ± 11
	Δ 95% CI	6 – 25	6 – 21	3 – 14	3 – 18	21 – 63	27 – 91	4 – 28
	*p*-value	0.005	0.0008	0.004	0.006	0.004	0.003	0.06
CG (*n* = 6)	PRE	75 ± 33	54 ± 18	47 ± 13	59 ± 14	77 ± 23	156 ± 64	85 ± 22
	POST	97 ± 39	70 ± 17	59 ± 13	74 ± 15	123 ± 37	278 ± 45	109 ± 16
	Δ	21 ± 8	16 ± 6	11 ± 3	14 ± 4	45 ± 20	54 ± 19	23 ± 14
	Δ 95% CI	13 – 30	10 – 23	7 – 16	10 – 20	24 – 67	34 – 76	8 – 39
	*p*-value	0.0005	0.0002	0.0005	0.0006	0.003	0.006	0.007
WG (*n* = 6)	PRE	63 ± 30	52 ± 26	47 ± 17	58 ± 16	81 ± 30	160 ± 51	87 ± 34
	POST	86 ± 37	65 ± 28	59 ± 17	72 ± 22	120 ± 49	212 ± 80	114 ± 41
	Δ	22 ± 10	13 ± 3	12 ± 3	15 ± 5	39 ± 24	51 ± 41	27 ± 11
	Δ 95% CI	1 – 34	9 – 16	9 – 16	10 – 20	13 – 65	8 – 95	15 – 39
	*p*-value	0.0004	0.001	0.0003	0.001	0.007	0.008	0.003
Interaction *p*-value		0.4	0.6	0.4	0.5	0.9	0.9	0.4

1 RM, one repetition maximum. Values are presented as mean ± standard deviation (SD), with all delta values (Δ) and 95% confidence interval from delta values (Δ 95% CI)). Interaction *p*-value, 3 groups (PG, CG, and WG) × 2 times (PRE and POST).

**Table 5:** Body composition before (PRE) and after (POST) an 8-week resistance training program in the placebo (PG), chia flour (CG) and whey protein (WG) group.

Groups		BW (kg)	FM (kg)	FM (%)	FFM (kg)
PG	PRE	65.2 ± 6.2	12.2 ± 5.6	19 ± 7.0	50.2 ± 3.3
(*n* = 6)	POST	66.9 ± 5.7	12.4 ± 5.4	18.9 ± 7.0	51.7 ± 2.8
	∆	1.7 ± 1.8	0.2 ± 1.6	−0.2 ± 2.2	1.5 ± 0.8
	∆ 95% CI	−0.3 − 3.6	−1.4 − 1.8	2522	0.7 − 3
	*p*-value	0.08	0.90	0.96	0.009
CG	PRE	66.3 ± 6.4	11.3 ± 3.7	17.7 ± 5.0	52.1 ± 5.4
(*n* = 6)	POST	66.7 ± 6.5	10.6 ± 3.7	16.4 ± 4.8	53.8 ± 4.4
	∆	0.4 ± 1.2	−0.7 ± 0.8	−1.4 ± 1.4	1.7 ± 1.3
	∆ 95% CI	−0.8 − 1.7	−1.6 − 0.2	−2.9 − 0.1	0.3 − 3.1
	*p*-value	0.9	0.5	0.16	0.003
WG	PRE	68.3 ± 8.4	14.0 ± 5.7	20.9 ± 6.9	51.3 ± 5.1
(*n* = 6)	POST	68.9 ± 8.1	13.5 ± 4.9	20.1 ± 6.3	52.5 ± 5.7
	∆	0.6 ± 2.0	−0.6 ± 1.2	−0.9 ± 0.9	1.2 ± 0.0
	∆ 95% CI	−1.5 − 2.6	−1.8 − 0.7	−2.0 − 0.1	0.2 − 2.1
	*p*-value	0.8	0.6	0.47	0.04
Interaction *p*-value		0.4	0.4	0.4	0.7

BW, body weight; FM, fat mass; FFM, fat free mass; Values are presented as mean ± standard deviation (SD), with all delta values (∆) and 95% confidence interval from delta values (∆ 95% CI) Interaclion *p*-value, 3 groups (PG, CG and WG) × 2 times (PRE and post).

## Data Availability

Data are available upon reasonable request to the corresponding author.
